# Robust Looming Spatial Localization in Dim Light via Daubechies Wavelet-Fused ON/OFF Pathways

**DOI:** 10.3390/biomimetics11040244

**Published:** 2026-04-03

**Authors:** Zefang Chang, Guangrong Wu, Hao Chen, He Zhang, Hao Luan, Zhijian Yang

**Affiliations:** 1 Institute for Math & AI, Wuhan, Wuhan University, Wuhan 430072, China; zfchang@whu.edu.cn; 2 School of Mathematics and Computational Science, Huaihua University, Huaihua 418099, China; 3 School of Mathematics, Sun Yat-sen University, Guangzhou 510275, China; 4 School of Mathematics and Information Science, Guangzhou University, Guangzhou 510006, China; 5 School of Automation and Electrical Engineering, Tianjin University of Technology and Education, Tianjin 300222, China

**Keywords:** dim light, spatial localization, Daubechies wavelet, ON/OFF streams, MLG1

## Abstract

Computational models of the MLG1 neurons in crab Neohelice granulata have been developed to detect and spatially localize looming stimuli. However, existing models suffer from significant performance degradation in dim scenarios, primarily due to visual signal corruption from stochastic noise such as photon shot noise. To address this challenge, we propose a computational framework that embeds Daubechies wavelet directly into ON/OFF visual pathways. The ON/OFF mechanism separates the input signals in parallel based on luminance changes to capture dynamic differences between target and background. Embedding Daubechies wavelet enables multi-scale frequency decomposition, allowing the model to suppress high-frequency noise while enhancing low-frequency looming trends. This process extracts low-frequency components and high-frequency details, providing the MLG1 neuron with more discriminative feature inputs. Experimental results demonstrate that the model achieves reliable looming spatial localization under extremely low contrast conditions, offering a robust methodology for bionic vision in extreme dim light environments.

## 1. Introduction

Vision-based collision perception is crucial for autonomous mobile systems [1,2]. However, mainstream methods heavily rely on specific sensors [3,4], limiting their deployment in power-constrained micro-robots. To find efficient and low-power solutions, researchers have turned to bio-inspired visual models. While the well-studied locust LGMD neuron provides efficient collision perception potential [5,6,7], it inherently lacks looming spatial localization capabilities. It can detect an approaching stimulus but cannot tell its exact direction, preventing robots from executing targeted evasive maneuvers. To bridge this gap, recent studies have modeled crab’s MLG1 visual system [8]. By using a population coding architecture of 16 MLG1 neurons covering a 360° field of view, this system successfully integrates both collision detection and spatial localization. However, despite these architectural advantages, existing MLG1-based models encounter a critical challenge in real-world deployment: they suffer from severe performance degradation in dim light environments.

The root cause of aforementioned failure lies in severe degradation of spatiotemporal transmission of motion information under dim light. This degradation is driven by two converging noise sources that fundamentally corrupt the signal in both space and time. First, the quantum nature of photons introduces a stochastic effect that dominates image formation process in photon-starved environments. Because photon arrival follows a Poisson distribution, the relative magnitude of photon shot noise—which is proportional to the square root of incident photon count—increases as the light level drops. In the spatiotemporal domain, this manifests as rapid, random fluctuations in pixel intensity over time, effectively masking the subtle grayscale gradients produced by a looming object. Second, to compensate for underexposure, the imaging hardware must utilize high circuit gain, which inevitably amplifies internal electronic noise. When these two noise components are combined, they saturate spatiotemporal signals with high-frequency interference. This noise "floor" forces pixel intensities to deviate from their true signal distribution, not only obscuring local textures but also invalidating detection methods that rely on regional grayscale variance to distinguish a moving target from background [9]. Because MLG1 system depends on the precise integration of motion cues across space and time, this total corruption of spatiotemporal signal prevents the accurate extraction and tracking of critical looming cues. Consequently, moving beyond traditional spatial-intensity domain represents a crucial necessity for breaking through current performance barriers in bio-inspired vision.

In fact, researchers have already turned their attention to the frequency domain to explore novel mechanisms for motion perception. Existing studies have confirmed that visual motion perception in the frequency domain offers distinct advantages in interpreting spatiotemporal dynamic information. Through methods such as Fourier and wavelet transforms, this approach can reveal encoding principles of motion information along the frequency dimension [10,11]. These studies focused on specific visual motion detection tasks by exploring methods to identify appropriate basis functions in the frequency domain for signal representation, which validated effectiveness of motion information under such frequency-domain basis characterizations. The transition to the frequency domain is not merely a mathematical convenience but is deeply rooted in biological architecture of arthropod visual system. Evidence suggests that ON/OFF pathways function as sophisticated temporal filters; for instance, the first-order interneurons (LMCs) act as band-pass filters that decouple dynamic motion from static background noise [12]. By decomposing visual stimuli into parallel streams of brightness increments and decrements, these pathways perform a biological frequency-tuning operation that isolates relevant motion energy.

Inspired by this intrinsic frequency-tuning mechanism, we propose a computational framework that integrates the multi-scale properties of Daubechies wavelet [13,14,15] directly into ON/OFF streams. Rather than relying on original spatiotemporal intensity, which is easily corrupted by photon shot noise and circuit gain in dim light, our model leverages differential responses of ON/OFF channels to extract high-contrast motion cues. By embedding Daubechies wavelet into these pathways, we perform a multi-scale frequency-domain decomposition and feature reconstruction. This process enables the simultaneous suppression of high-frequency stochastic noise and the enhancement of low-frequency looming trends. The resulting features provide an MLG1-based spatial localization network with inputs of significantly higher discriminability than traditional intensity-based models. Finally, the proposed model is validated through numerical experiments across varying levels of illumination. Systematic results demonstrate that by synthesizing biological contrast encoding with mathematical frequency localization, our model achieves reliable motion detection and precise spatial localization in extreme dim light environments.

The main contributions of this paper are summarized as follows:

- To address the limited polarity-specific sensitivity of MLG1 model to approaching threats, we introduce novel neural computing architecture that incorporates an ON/OFF mechanism into a looming spatial localization visual system.
- This bio-inspired modeling research introduces a novel paradigm for dim light conditions, employing Daubechies wavelet neural network based on ON/OFF channels to address the challenge of looming spatial localization.
- This paper explores the specific implementation of MLG1 modeling combined with multi-scale frequency analysis in the topic of brain-inspired visual perception, extending the broader field of bio-inspired dim light vision systems. This work contributes novel perspectives to applications in specific domains such as dim light robotic navigation and autonomous collision avoidance.

This paper is organized into five sections. Section 2 reviews the biological background of dim light vision. Section 3 introduces the proposed model framework and Daubechies wavelet. Section 4 presents and analyzes the experimental findings. The paper concludes with a summary in Section 5.

## 2. Biological Mechanisms of Dim Light Vision

In dim light environments, animals face the constraints of photon scarcity and intrinsic noise, which generally impair visual discrimination. To overcome this challenge, their visual systems have evolved synergistic adaptive mechanisms. These mechanisms are founded on specialized photoreceptor structures, centered on efficient integration of neural signals and supported by precise noise regulation. Furthermore, divergent ecological niches and behavioral demands have driven the development of diverse, specialized strategies across different species.

Overall, most animals enhance visual sensitivity at the cost of spatiotemporal resolution [16,17,18,19,20,21]. They typically rely on specialized photoreceptors for scotopic vision, such as vertebrates’ rod cells [22,23,24,25] or the compound eyes of insects [18]. To integrate faint light signals, visual systems prolong temporal summation, for instance with integration times of about 0.1 s [22,25] in humans, 1.5–1.9 s [26,27] in toads and up to 220 ms [28] in moths. They also employ spatial summation, expanding receptive fields to gather signals over wider areas, such as approximately 0.4 square degrees in humans [22,25] or across 18 ommatidia in the L-fibers of bees [18,21]. Regarding noise control, inherent disturbances like spontaneous rhodopsin [22,23,24,25] activation necessitate cooperative activation across multiple photoreceptors or population-level neural coding to reliably distinguish genuine signals from background noise. Collectively, these adaptations improve detection capability but also lead to common consequences of reduced spatial acuity and slower response speeds in dim light.

Evolutionary paths to dim light vision are highly divergent across taxa. Humans and mammals favor stable signal detection, augmented by neural strategies like collective and predictive coding in ganglion cells to boost the overall signal-to-noise ratio for discerning environmental details [24]. Amphibians, however, have evolved a dual-rod system capable of color discrimination in dim light; in frogs, this allows for distinguishing blue from green, with performance thresholds that plummet to starlight levels during crucial behaviors like foraging [29]. Insects such as moths and bees not only preserve color constancy for flower identification [30] but also possess visual systems finely attuned to their lifestyle, bees for landmark-based homing [18,20,21,31] and moths for detecting slow motion [32].

In summary, visual discrimination in dim light is not merely degraded but is reconfigured through a series of targeted specializations. These adaptations effectively balance sensitivity with specificity, ultimately supporting critical survival behaviors such as foraging, predator avoidance and navigation.

Although the precise neural mechanisms remain unclear, these visual adaptations function as highly effective noise filters and signal enhancers in dim light. Inspired by this, we attempt to translate these visual adaptation mechanisms into a computational model. In the following section, we introduce a network architecture that combines crab’s MLG1 spatial localization system with Daubechies wavelet. This design aims to computationally emulate the noise-resilient ON/OFF pathways found in nature, bridging the gap between biological inspiration and engineering application.

## 3. Network Architecture

**Figure 1:**
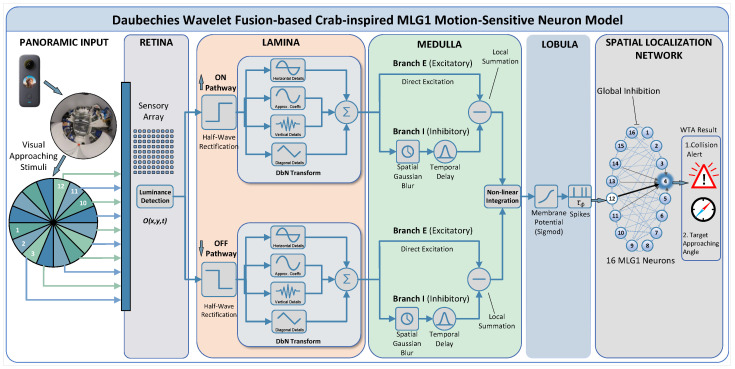
The full field-of-view image from panoramic camera is divided into 16 equal segments, each corresponding to an MLG1 neuronal unit acting as an independent local visual information perception module within the visual neural network, responsible for specialized processing of visual features within its respective region. Each ommatidium (unit in sensory array) first detects pixel-level local changes in luminance within its visual field and subsequently relays this signal to both ON and OFF pathways. Following Daubechies wavelet transformation, these signals undergo integration in the medulla layer through competitive interactions between excitatory and inhibitory units, ultimately converging onto the MLG1 neuron.

To replicate 360° panoramic visual receptive field of crab Neohelice granulata, we use a panoramic camera and equally divide its horizontal field of view into 16 independent processing sectors (22.5° each). As shown in Figure 1, an ON/OFF mechanism is introduced to process luminance increases and decreases independently. Moreover, we integrate Daubechies wavelet into both pathways to extract multi-scale features. This enables a robust approach to motion detection in dim light.

### 3.1. Daubechies Wavelet

Daubechies wavelet represents a highly influential family of orthogonal wavelets within the field of wavelet analysis [13,33]. Their defining characteristics are derived from an iterative construction method, which yields wavelets with compact support while simultaneously satisfying orthogonality conditions. Multi-scale analysis is the theoretical core of Daubechies wavelet transform. Its essence is to achieve hierarchical decomposition and characterization of signals through a set of nested function space sequences. In the initial stage of transform, the signal to be analyzed is decomposed into a low-frequency component and a high-frequency component. The low-frequency component, which captures signal’s outline or approximation, is referred to as approximation coefficient. The high-frequency component, which captures details and transients, is known as the detail coefficient. This decomposition process progressively reveals the features of signal at different scales.

The Daubechies wavelet family is commonly denoted as DbN, where N indicates the order of wavelet. It is worth noting that the Db1 wavelet is mathematically equivalent to Haar wavelet. Daubechies wavelet transform is of order N, employing filters of length 2N; that is,

(1) $$\phi \left(x\right)=\sqrt{2}\sum_{i=0}^{2N-1}{\left(-1\right)}^{i}h_{2N-1-i}\psi \left(2x-i\right)$$

where $h_{0},h_{1},\ldots ,h_{2N-1}$ are a set of constant filter coefficients that satisfy the following conditions:

(2) $$\sum_{i=0}^{N-1}h_{2i}=\frac{1}{\sqrt{2}}=\sum_{i=0}^{N-1}h_{2i+1}$$

As well, for *j* = 0, 1, …, *N* − 1.

(3) $$\sum_{i=0}^{2N-1+2j}h_{i}h_{2i-1}=\left\{\begin{array}{cc} 1 & \mathrm{if}\;j=0\\ 0 & \mathrm{if}\;j\neq 0 \end{array}\right.$$

Daubechies scaling function ψ is given by the following recursion equation:

(4) $$\psi \left(x\right)=\sqrt{2}\sum_{i=0}^{2N-1}h_{i}\psi \left(2x-i\right)$$

The central task in constructing Daubechies wavelet is to determine the filter coefficients $h_{0},h_{1},\ldots ,h_{2N-1}$. These 2N coefficients are subject to *N* + 2 constraint equations. As a concrete example, for *N* = 2, there are four coefficients: $h_{0},h_{1},h_{2},h_{3}$.

Once the scaling function φ is derived, one can compute the expansion coefficients $a_{i},d_{i}$ for a given function, which are yielded by the following inner products:

(5) $$a_{i}=\left(f,\varphi_{i}\right)=\int f\left(x\right)\bar{\varphi_{i}\left(x\right)}dx$$

(6) $$d_{i}=\left(f,\phi_{i}\right)=\int f\left(x\right)\bar{\phi_{i}\left(x\right)}dx$$

In this study, we use Daubechies wavelet to process visual signals. Its multi-scale features fit well with ON and OFF pathways of crab visual system. This mathematical method helps the model separate brightness changes and detect motion clearly in dim light.

### 3.2. Retina Layer

The numerous ommatidia located in retina layer function as luminance receptors, working in concert to acquire visual information from external environment. These ommatidia are arranged in matrix-like configuration, with each unit independently detecting instantaneous changes in brightness within its corresponding field of view, thereby discretizing the continuous optical world into a dynamic array of luminance signals (see Figure 1). Formally, the input of continuous visual stimulus is denoted as a spatiotemporal image stream I(x,y,t), where (x,y) represents spatial coordinates and *t* denotes time. This brightness acquisition process can be formally expressed as follows:

(7) $$O\left(x,y,t\right)=\int \left(\delta (t-\tau )-\delta (t-\Delta t-\tau )\right)I\left(x,y,\tau \right)d\tau$$

where δ is the unit impulse function.

### 3.3. Lamina Layer

The brightness variation detected by each ommatidium over time corresponds essentially to temporal derivative of luminance signal. Downstream neurons act as a biological half-wave rectifier, utilizing highly specialized synaptic mechanisms to precisely segregate mixed temporal derivative signals received from ommatidia into two parallel processing pathways, the ON and OFF channels. ON channel is characterized by its high specificity to luminance increments. Through a positive half-wave rectifier mechanism, it selectively extracts positive temporal derivative components, effectively filtering out all irrelevant information. In contrast, OFF channel is specialized to respond to negative temporal derivatives (signaling luminance decrements) and is computationally modeled as a negative half-wave rectifier. The specific implementation mechanism is described as follows:

(8) $${\tilde{O}}_{on}\left(x,y,t\right)={\left[O\left(x,y,t\right)\right]}^{+}$$

(9) $${\tilde{O}}_{off}\left(x,y,t\right)={\left[O\left(x,y,t\right)\right]}^{-}$$

where $O_{on}\left(x,y,t\right)$ and $O_{off}\left(x,y,t\right)$ are defined as signals resulting from the half-wave rectifier of the first layer’s output by ON and OFF channels, respectively. ${\left[O\right]}^{+}$ and ${\left[O\right]}^{-}$ represent max(O,0) and max(−O,0), respectively.

To balance rapid temporal response with accurate spatial feature extraction, Daubechies wavelet is introduced to optimize $\tilde{O}$ signals. Characterized by compact support and orthogonality, this class of wavelets enables joint time–frequency analysis of signals while preserving the low-latency properties of ON channel; that is,

(10) $$O_{on}\left(x,y,t\right)=DbN\left({\tilde{O}}_{on}\left(x,y,t\right)\right)$$

Specifically, output from the half-wave rectifier first undergoes a wavelet transform, decomposing ${\tilde{O}}_{on}\left(x,y,t\right)$ into four coefficient sub-bands, approximating coefficient (cA), along with the horizontal (cH), vertical (cV) and diagonal detail (cd) coefficients. Here, cA retains the overall trend of luminance increase, aligning with the core response characteristics of ${\tilde{O}}_{on}$ signal. The three sets of detail coefficients respectively capture localized spatial variations in the horizontal, vertical and diagonal directions—such as luminance jumps at edges and fine textural details—thereby supplementing spatial information absent in original output. To highlight salient features prioritized by the visual system, a weighted fusion of detail coefficients is performed. In accordance with ON channel’s sensitivity to luminance increment features, the following weighting formula is applied:

(11) $$Coeff_{on}=0.3|cH|+0.3|cV|+0.1|cD|+0.3|cA|$$

Daubechies wavelet transform is applied to ${\tilde{O}}_{off}$ signal; that is,

(12) $$O_{off}\left(x,y,t\right)=DbN\left({\tilde{O}}_{off}\left(x,y,t\right)\right)$$

The transformation yields four sets of coefficients: cA, cH, cV and cD. Based on these, the feature coefficients for OFF channel are derived from a weighted fusion of the detail coefficients. This process facilitates selective extraction and integration of spatial detail features in the wavelet domain, as defined by the following equation:

(13) $$Coeff_{off}=0.3|cH|+0.3|cV|+0.1|cD|+0.3|cA|$$

### 3.4. Medulla Layer

In ON channel signal processing, excitatory cells directly transform the positive time-derivative signals of luminance received from photoreceptors into excitatory outputs with negligible delay; that is,

(14) $$E_{on}\left(x,y,t\right)=O_{on}\left(x,y,t\right)$$

where $E_{on}\left(x,y,t\right)$ is the excitation signal.

The inhibitory circuitry in ON channel serves to laterally integrate signals from multiple $O_{on}$ cells, generating delayed inhibitory output that regulates excitatory activity. This integration mechanism, essential for processing spatial information, consequently introduces a significant latency compared to the direct pathway; that is,

(15) $$\begin{array}{c} I_{on}\left(x,y,t\right)=\;\int \int \int \;O_{on}\left(u,v,\tau \right)\Phi \left(t-\tau \right)\times \\ G_{\sigma }\left(x-u,y-v\right)dudvd\tau \end{array}$$

where $I_{on}\left(x,y,t\right)$ represents the output of inhibitory unit within ON channel. $G_{\sigma }\left(x,y\right)$ is a Gaussian function, defined as

(16) $$G_{\sigma }\left(x,y\right)=\frac{1}{2\pi \sigma^{2}}e^{\left(-\frac{x^{2}+y^{2}}{2\sigma^{2}}\right)}$$

where σ is the standard deviation of Gaussian function.

Within the model, the delay is implemented by filter Φ(t), formulated as

(17) $$\Phi \left(t\right)=2e^{-\pi t^{2}}$$

Rather than linear summation, the integration of excitatory and inhibitory signals in the local summation unit is implemented by a positive half-wave rectification. This operation filters out any negative net input; that is,

(18) $$Ls_{on}\left(x,y,t\right)={\left[E_{on}\left(x,y,t\right)-\varsigma \cdot I_{on}\left(x,y,t\right)\right]}^{+}$$

where $Ls_{on}\left(x,y,t\right)$ represents the local summation unit. The inhibitory contribution is modulated by a coefficient ς.

OFF channel processes signals in a manner analogous to the ON channel. Its excitatory cells provide an immediate response to luminance increments at the center, while delayed inhibitory cells deliver suppressive signals from surroundings. When a moving object appears in the visual field, excitatory cells are activated first. As object moves away, the surrounding region it previously occupied subsequently triggers inhibitory cells, generating a delayed inhibitory response, that is,

(19) $$E_{off}\left(x,y,t\right)=O_{off}\left(x,y,t\right)$$

(20) $$\begin{array}{c} I_{off}\left(x,y,t\right)=\;\int \int \int \;O_{off}\left(u,v,\tau \right)\Phi \left(t-\tau \right)\times \\ G_{\sigma }\left(x-u,y-v\right)dudvd\tau \end{array}$$

where $E_{off}\left(x,y,t\right)$ and $I_{off}\left(x,y,t\right)$ denote OFF channel’s excitatory and inhibitory components, respectively.

In the local summation unit, motion information is initially extracted by computing difference between the central excitatory signal and surrounding inhibitory signal. This difference is then passed through a half-wave rectifier that sets all negative values to zero. This ensures the system responds only to motion in a specific direction, thereby encoding the polarity of movement; that is,

(21) $$Ls_{off}\left(x,y,t\right)={\left[E_{off}\left(x,y,t\right)-\varsigma \cdot I_{off}\left(x,y,t\right)\right]}^{+}$$

where $LS_{off}\left(x,y,t\right)$ denotes the local summation unit of OFF channel. ς is coefficient controlling the strength of inhibitory stream.

### 3.5. Lobula Layer

At the summation stage of visual signal processing, local signals from ON and OFF channels are no longer maintained as purely parallel pathways. Instead, they are merged through an integration strategy to achieve a synergistic fusion of luminance change information with opposite polarities. This process is described as

(22) $$\begin{array}{c} S\left(x,y,t\right)=\alpha_{1}Ls_{on}\left(x,y,t\right)+\alpha_{2}Ls_{off}\left(x,y,t\right)\\ +\alpha_{3}Ls_{on}\left(x,y,t\right)Ls_{off}\left(x,y,t\right) \end{array}$$

where $\alpha_{i},i=1,2,3$ are used to describe cooperative correlation characteristics between the ON/OFF channel signals.

Subsequently, all excitatory inputs are linearly integrated to form aggregate postsynaptic potential. This potential is then transformed by an exponential function into the final output membrane potential. This transformation not only confines output within a physiologically plausible range for membrane potential but also introduces a essential nonlinearity, enabling the network to represent complex, nonlinear mappings between its inputs and outputs; that is,

(23) $$k_{Mp}\left(t\right)=\int_{1}^{R}\int_{1}^{C}S\left(x,y,t\right)dxdy$$

(24) $$Mp\left(t\right)=\frac{1}{1+e^{-k_{Mp}\left(t\right)\cdot {\left(C\cdot R\cdot \mu \right)}^{-1}}}$$

where *C* and *R* represent the width and height of input image sequence, respectively. μ is a scaling parameter.

The output membrane potential is mapped to spikes via an exponential function; that is,

(25) $$S_{sp}\left(t\right)=\left[e^{\left(\nu \left(K_{Mp}\left(t\right)-T_{sp}\right)\right)}\right]$$

In this framework, ν serves as a scaling parameter that governs the firing rate and $T_{sp}$ defines spike threshold. During real-time robotic experiments, collision alerts are issued according to the following expression:

(26) $$FC_{A}=\left\{\begin{array}{cc} 1 & \mathrm{if}\;\sum_{i=t-t_{n}}^{t}S_{sp}\left(i\right)\geq n_{sp}\\ 0 & \mathrm{otherwise} \end{array}\right.$$

where $n_{sp}$ denotes the number of spikes in a specified time window constituted by $t_{n}$ successive digital signal frames.

After that, target localization in adjacent spatial positions is achieved through a winner-takes-all (WTA) network. This WTA circuit comprises 16 MLG1 neurons, a hidden layer and a latent spatial localization (LSL) layer, with the LSL layer incorporating both excitatory and inhibitory synaptic connections. Signal modulation in the hidden layer follows a rule of specific excitation and global inhibition: each neuron in this layer receives excitatory input from its corresponding MLG1 neuron, while being subject to inhibitory regulation from other MLG1 neurons. Specifically, when a given MLG1 neuron fires a spike at time t, the corresponding hidden neuron is rapidly activated, while all other hidden neurons in the network are simultaneously suppressed. This competitive mechanism serves to highlight the neural response associated with target location [8].

### 3.6. Algorithm Summary

To make our computational framework easy to understand, we show the complete processing steps in Algorithm 1. It lists all computing stages, from taking the input video in retina layer to sending final collision alert in spatial localization network.   
**Algorithm 1:** Online algorithm of proposed model
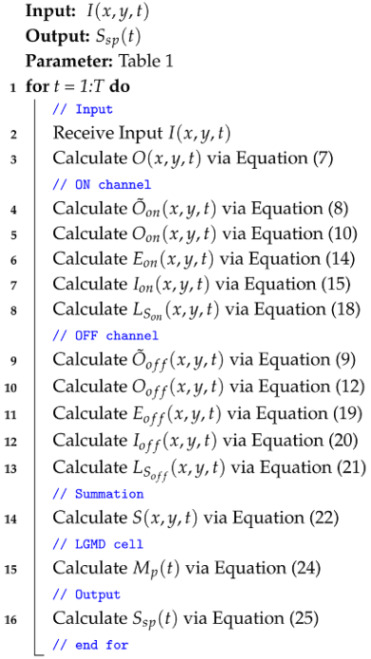


## 4. Experimental Results and Analysis

### 4.1. Experimental Setup

To ensure authenticity of experiments and reliability of conclusions, all image sequences used in this study are captured and processed from real-world scenes. These sequences are recorded using the Insta360 ONE X panoramic camera, whose wide-angle shooting capability and flexible viewing angle adjustment effectively simulate the panoramic visual perception mode of a crab compound eye. All experimental videos have a resolution of 720 × 720. The parameter configurations of the proposed visual perception model are summarized in Table 1.

To achieve a comprehensive assessment, this study employs a combination of two contrast metrics to mitigate limitations inherent in relying on a single one. Root Mean Square Contrast (RMS) is quantified by calculating standard deviation from mean pixel intensity. It is calculated as follows:

(27) $$C_{RMS}=\sqrt{\frac{1}{N}\sum_{i=1}^{N}{\left(x_{i}-\bar{x}\right)}^{2}}$$

where $x_{i}$ represents intensity value of the *i*-th pixel, $\bar{x}$ denotes mean intensity value across all pixels, and *N* is the total number of pixels. Another indicator is

(28) $$C_{M}=\frac{|I_{max}-I_{min}|}{255}$$

where $I_{max}$ and $I_{min}$ are defined as the maximum and minimum pixel intensity of image, respectively.

### 4.2. Detection of Looming Spatial Location

To systematically validate effectiveness of the proposed model in dynamic detection tasks, we conduct comparative experiments between our model and the MLG1 model [8]. The analysis focuses on membrane potential output characteristics of 16 core functional modules in both types of models. Based on this analysis, we quantitatively evaluate recognition effectiveness of the proposed model for localization events.

**Figure 2:**
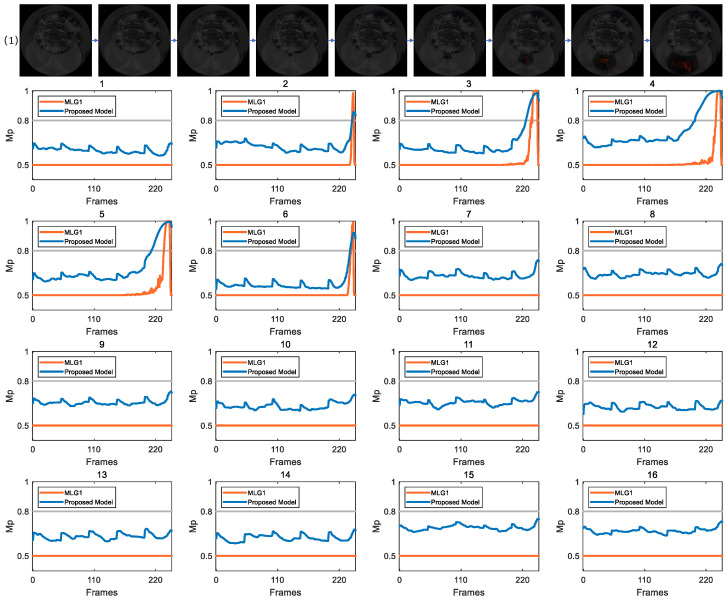
In this scenario, $C_{\mathrm{RMS}}$ = 15.35 and $C_{M}$ = 0.13. Both the MLG1 model and proposed model exhibit membrane potentials that exceed threshold in modules 2 through 6. In the remaining modules, the membrane potentials do not reach threshold, which was set to 0.8 in all experiments.

In the image sequences show in Figure 2, the parameter $C_{RMS}$ is 15.35 and $C_{M}$ is 0.13. This combination indicates moderate overall luminance fluctuation and a distinct grayscale difference between target and background, thereby providing favorable conditions for these models to effectively extract key visual features such as edges and contours. The figure clearly illustrates dynamic membrane potential curves of 16 functional modules in two models across the image frame sequence. Under an experimental condition where exceeding a preset warning threshold triggers collision alert, the curves reveal that only a small subset of modules generated effective responses to the collision stimulus. As revealed by membrane potential response results presented in the figure, the proposed model and MLG1 model demonstrate highly consistent response patterns under the given experimental conditions. Specifically, both models incorporate five core functional modules (numbered 2–6), whose membrane potential variation curves exceed collision warning threshold. In contrast, the remaining 11 functional modules exhibit no significant activity during the experiment, as their membrane potentials consistently remain below triggering threshold throughout the entire image sequence. In Table 2, we have listed the contrast and luminance values of experimental scene, as well as the number of frames in which each model first responded to the membrane potential for comparison. This key observation indicates that, supported by visual inputs with relatively high luminance, both models possess the fundamental capacity to reliably capture collision signals. Among the five functional modules, module 4 demonstrates the highest sensitivity, as its membrane potential crosses warning threshold significantly earlier than those of other modules. According to the winner-takes-all decision rule, module 4 gains decision priority due to its temporal advantage. The corresponding visual receptive field of this module is thereby identified as the core spatial region of collision stimulus, enabling precise localization of collision source.

### 4.3. Multiple Size and Looming Velocity Tests

**Figure 3:**
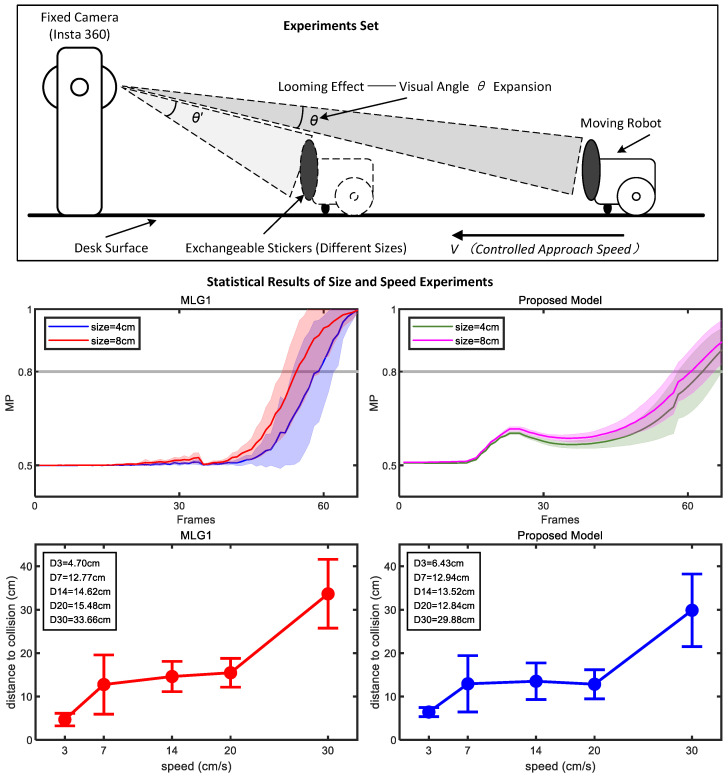
Two objects of different sizes are used as looming stimuli. Statistical analysis (shaded error bars) reveals that neural responses occur earlier in response to the larger object relative to the smaller one. The experiment employs five looming stimuli with graded approach speeds. To mitigate the interference of random errors, each speed condition is independently repeated five times. Results show that across all comparative experimental groups, the warning distance of two models exhibit a significant increasing trend as target’s approach speed rises. The looming stimulus with the highest speed corresponds to the longest warning distance.

To validate the proposed model’s ability to perceive object size, we conduct comparative experiment systematically examining effect of looming target size on the model’s output. In the experiment, black stickers with diameters of 4 cm and 8 cm are attached by robots and used as approaching stimuli. Previous physiological studies have demonstrated that a crab’s MLG1 neuron exhibits a clear size-dependent response pattern: the larger the physical size of an approaching target, the shorter the latency of the neuron’s spike generation, meaning neural response occurs earlier. The middle coordinate graph in Figure 3 presents a comparison of dynamic membrane potential responses between the proposed model and MLG1 model. Under stimulation with targets of 4 cm and 8 cm in diameter, the time at which membrane potential exceeds threshold is significantly earlier for the 8 cm target than for the 4 cm target. The membrane potential rise trajectory and spike timing of the models show high degree of consistency with physiological recordings from the MLG1 neuron. Importantly, the proposed model accurately reproduces the key feature that a larger target size leads to shorter response latency. These results fully demonstrate that the proposed model not only replicates response patterns of the MLG1 neuron at physiological level but also aligns quantitatively with perceptual characteristics of the biological visual system in terms of size coding.

Furthermore, this study further investigates the influence of approaching speed of looming object on the distance to collision (DTC). In the experiments, five graded speed levels (3 cm/s, 7 cm/s, 14 cm/s, 20 cm/s and 30 cm/s) are selected to cover typical collision scenarios ranging from low to high speeds. Each speed condition is repeated 10 times to minimize random error (as shown in the bottom coordinate graph in Figure 3). Statistical analysis reveals that both the proposed model and MLG1 model exhibit significant speed-dependent variation in DTC variation; as approaching speed of object increases, the DTC of both models shows monotonic increasing trend. Specifically, under the low-speed condition of 3 cm/s, the average DTC of proposed model over 10 repeated trials is 6.43 cm, while that of the MLG1 neuronal model is 4.70 cm. When approaching speed rises to 30 cm/s, the average DTC of proposed model increases significantly to 29.88 cm and that of MLG1 model rises to 33.66 cm. This trend is highly consistent with existing biological findings, indicating that the faster object approaches, the greater the DTC at which MLG1 neuron triggers collision avoidance behavior.

In biology, this DTC is exactly like the Flight Initiation Distance (FID) of escaping animals. When a predator approaches faster, the crab starts to run away earlier to keep a safe distance. Our experiment shows that the proposed model successfully imitates this biological survival skill.

### 4.4. Results Under Testing with Structured Indoor Scenes

**Figure 4:**
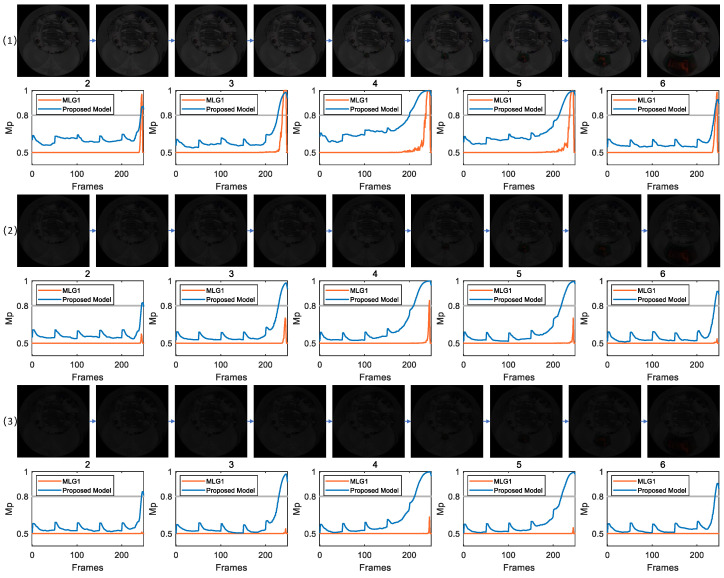
$C_{\mathrm{RMS}}$ of scenarios (1), (2) and (3) are 14.28, 7.65 and 6.00, respectively. For the MLG1 model, when the $C_{RMS}$ value is 6.00 (i.e., scenario (3)), the membrane potential of all its modules fails to exceed threshold, leading to an inability to achieve motion detection and localization. The proposed model is capable of stably achieving motion localization across all three scenarios.

Figure 4 presents experiments conducted under gradually decreasing indoor illumination. This setup enables a systematic comparison of collision detection responses between the proposed model and MLG1 model. The study aims to examine the performance stability and adaptability of both models as brightness continuously diminishes, thereby providing more dynamic evidence for evaluating robustness of the models. For clarity, the figure displays only membrane potential curves from modules that exceed the warning threshold, thereby omitting redundant data from silent modules to emphasize critical response features.

Figure 4 (1) displays results under conditions with a $C_{RMS}$ of 14.28 and $C_{M}$ of 0.32. At this level, the target’s contours and motion features in visual input remain clearly distinguishable. The results show consistent response patterns between the proposed model and MLG1 model: modules 2, 3, 4, 5 and 6 exceed warning threshold, with no missing or erroneous responses observed. It is worth noting that membrane potentials of the proposed model and MLG1 model in module 4 exceed threshold at frames 201 and 236, respectively. By applying the winner-takes-all decision principle and considering MLG4’s earlier initial response, the core spatial region of emerging collision stimulus is determined to lie within the receptive field of the MLG4 module. These results confirm that both models achieve accurate collision identification and spatial localization under medium–high contrast conditions.

In Figure 4 (2), contrast parameters are further reduced to $C_{RMS}$ = 7.65 and $C_{M}$ = 0.17, simulating an indoor environment with significantly degraded visual input. The results reveal a clear divergence in model performance: the proposed model maintains a stable response, while membrane potential of the MLG1 model only exceeds threshold in module 4. This performance gap indicates that feature extraction capability of the proposed model in dim light environments is superior to that of the MLG1 model.

In Figure 4 (3), an extremely dim light setting ($C_{RMS}$ = 6.00 and $C_{M}$ = 0.13) is used to simulate a scenario where target features are nearly indistinguishable. The experimental data further validate robustness of the proposed model. In contrast, the MLG1 model continues to fail, mirroring its performance in the preceding dim light scenario.

**Figure 5:**
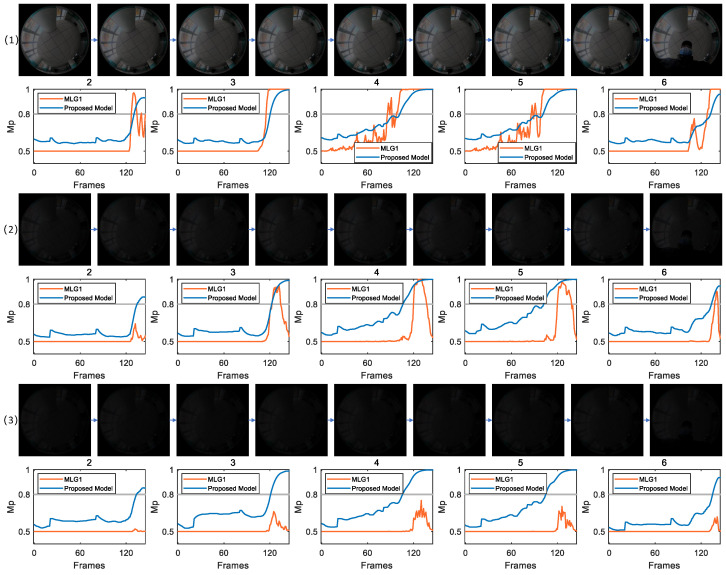
This figure also presents three scenarios with varying brightness levels. As brightness gradually weakens, the membrane potential of the MLG1 model is significantly affected.

Figure 5 compares the collision detection responses of the proposed and MLG1 models to different visual stimuli in an identical indoor scene. Figure 5 (1) corresponds to experimental conditions with $C_{RMS}$ = 31.07 and $C_{M}$ = 0.48 for the image sequence. In this scenario, target features are obvious, which helps the models in extracting valid visual information. The results demonstrate a consistent response pattern between the proposed model and MLG1 model. In both models, membrane potentials of five functional modules (2, 3, 4, 5, and 6) exceed the preset warning threshold, indicating that each model is capable of detecting collision signals when provided with high-quality visual input. Analysis of the response dynamics reveals that module 5 is the most sensitive, as evidenced by the earliest threshold crossing. By applying the winner-takes-all rule, this temporal activation pattern allows the core spatial region of collision stimulus to be pinpointed to the module 5 receptive field. Consequently, these findings validate the reliable collision recognition and localization abilities of both models when visual contrast is sufficiently high.

For Figure 5 (2), the contrast parameters of image sequence are reduced to $C_{RMS}=7.82$ and $C_{M}=0.13$. Since the same scene is used and only brightness is changed without altering the spatial attributes or motion characteristics of target, it is theoretically expected that the response module set will be consistent with Figure 5 (1); that is, modules 2, 3, 4, 5 and 6 should exhibit membrane potentials above the threshold. The proposed model exhibits a membrane potential response characteristic that aligns closely with the trend shown in Figure 5 (1). Specifically, its module 5 demonstrates a significant response, with its membrane potential being the first to exceed threshold at frame 103. In contrast, the MLG1 model shows notably weaker activation: only four of its functional modules reach threshold level and the amplitude of membrane potential in each activated module is significantly attenuated compared to that in Figure 5 (1). Nevertheless, module 5 of the MLG1 model retains its core localization function, surpassing threshold at frame 120, and displays target preference consistent with that of the proposed model.

Figure 5 (3) presents experimental results under an extremely dim light condition ($C_{RMS}=5.40$ and $C_{M}=0.09$). In this scenario, significant noise severely weakens visual edges and motion features of the collision target, which poses a substantial challenge to robustness of feature extraction and sensitivity of signal identification. Although the experimental scene remains unchanged and the same set of modules (modules 2, 3, 4, 5, and 6) is theoretically expected to respond, the actual performance of two models diverges markedly. The proposed model maintains stable performance under these extreme conditions: all five key modules successfully exceed warning threshold, accurately triggering collision alert, and the stimulus is correctly localized to the receptive field of module 5. In contrast, the MLG1 model fails completely, with none of its modules producing membrane potential above warning threshold. Consequently, it generates no effective collision response, failing to both detect the collision event and localize the target.

The scenarios with varying brightness levels in figures demonstrate that, under these challenging conditions, our model overcomes performance limitations of the MLG1 model and achieves reliable collision recognition and localization in extremely dim light environments.

### 4.5. Results Under Testing of Complex Dynamic Scenes

**Figure 6:**
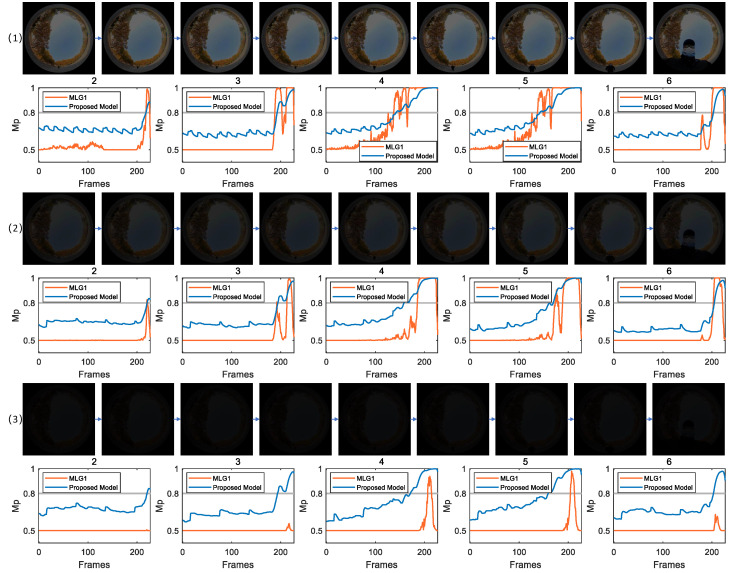
These are three scenarios captured outdoors. As brightness weakens, the membrane potential of the proposed model is not significantly affected; it can still achieve motion detection even in a dim light scenario (i.e., scenario (3)).

Figure 6 presents a comparative analysis of collision detection and localization performance between the proposed model and MLG1 model in outdoor complex dynamic scenes under varying levels of illumination. The experiments are designed based on natural environmental challenges such as fluctuating illumination and background interference. By progressively reducing the contrast of image sequences in a gradient manner, the robustness of both models is evaluated under extreme visual conditions.

In the image sequence corresponding to Figure 6 (1), $C_{RMS}$ and $C_{M}$ are 35.83 and 0.51, respectively. As shown in the figure, modules 2, 3, 4, 5 and 6 in both the proposed model and MLG1 model exhibit membrane potentials that exceed threshold. Module 5 is the first in which membrane potentials exceed threshold. According to the winner-takes-all principle, the core receptive field corresponding to looming stimulus is localized to module 5. These experiments demonstrate that under relatively high illumination levels, both models effectively capture motion features and achieve accurate collision detection and localization.

Figure 6 (2) uses an outdoor scene image sequence from the same source as (1), with its $C_{RMS}$ reduced to 13.33 and $C_{M}$ further lowered to 0.20, simulating visual signal degradation due to diminished illumination. Theoretically, the spatial distribution characteristics of identical collision stimulus remain unchanged; thus, activated functional modules should correspond to those in (1) with membrane potentials expected to reach the activation threshold. Experimental results reveal that the proposed model and MLG1 model maintain stable responses; all five modules are effectively activated and looming stimulus is localized to the receptive field corresponding to module 5.

Figure 6 (3) shows the results for dim light condition, with $C_{RMS}$ and $C_{M}$ as low as 4.63 and 0.07, respectively, simulating the limit of visual perception under dark conditions. Theoretically, the spatial characteristics of collision stimulus are consistent with those of the previous two groups of experiments; thus, modules 2, 3, 4, 5 and 6 should be activated. However, the actual results reveal notable discrepancy. Leveraging Daubechies wavelet-optimized signal processing mechanism embedded in its ON/OFF channels, the proposed model successfully drives membrane potentials above threshold in five modules under this dim light scenario, thereby achieving effective collision warning. In stark contrast, the MLG1 model fails to activate any of the relevant functional modules: it neither identifies collision risks nor locates the moving object. These findings further validate the superior performance of the proposed model in extremely dim light environments.

**Figure 7:**
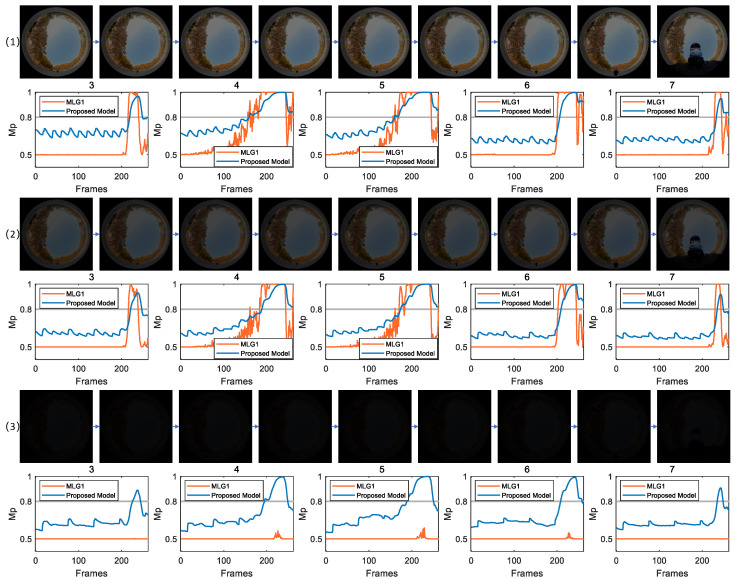
$C_{RMS}$ of scenarios (1), (2) and (3) are 40.65, 23.33 and 3.48, respectively. The proposed model is capable of achieving motion localization across all three scenarios.

Figure 7 displays a set of collision scenes captured outdoors. In (1), $C_{RMS}=40.65$ ($C_{M}=0.58$). As clearly shown in Figure 7 (1), the effective membrane potential responses of both the proposed model and MLG1 model are specifically concentrated in five consecutive spatial perception modules, numbers 3, 4, 5, 6 and 7, confirming consistency in spatial response characteristics between two types of models. In terms of response timing, both models exhibit the first membrane potential threshold-crossing behavior in module 5. Specifically, membrane potential signal of the MLG1 model reaches the preset activation threshold at frame 156, while the proposed model achieves threshold crossing at frame 160. According to the winner-takes-all localization principle, both models locate pedestrian’s core position in this scene at spatial coordinates corresponding to module 5. The membrane potential response curves of two models in Figure 7 (2) are similar to those in Figure 7 (1).

In Figure 7 (3), $C_{\mathrm{RMS}}=3.48$ and $C_{M}=0.06$. Compared with the scenes in other subfigures, the values of $C_{\mathrm{RMS}}$ and $C_{M}$ are markedly lower, indicating significantly weaker target-related motion signals in this scenario. As illustrated, the proposed model demonstrates strong adaptability to dim light conditions. The membrane potential response curves in its spatial perception modules 3, 4, 5, 6 and 7 consistently exceed threshold. Among these, module 5 is again the first to cross threshold, further confirming the spatial localization stability of the proposed model. Regardless of signal strength, its core response remains consistently focused on key spatial modules. In contrast, the MLG1 model fails to generate any effective response under the same conditions. It is unable to adequately extract weak motion signals from complex background, resulting in a complete loss of localization capability in such dim light scenarios. Taken together, these results collectively confirm the distinct advantage of the proposed model in handling signal processing challenges presented by dim light environments.

**Figure 8:**
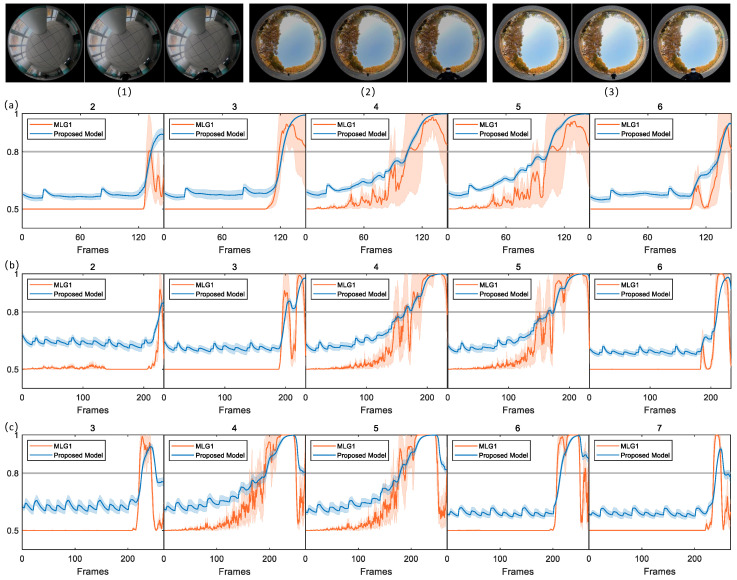
This figure presents the statistical chart of membrane potential variance for the two models across three scenarios. Evidently, the membrane potential output of the proposed model remains highly stable even as brightness decreases.

To validate the performance stability of the proposed model under varying illumination conditions, this study designs a multi-scenario comparative experiment. The testing covers one indoor scenario and two typical outdoor scenarios. For each scenario, 20 sub-scenarios with different illumination intensities are randomly selected for evaluation. The contrast ratio ($C_{M}$) of all test samples ranges from 0.08 to 0.60, covering typical conditions from low and dim light to normal illumination, thereby ensuring the generalizability of the experimental results. Figure 8 presents the statistical results of membrane potential variance output by the models across the test scenarios. Only membrane potential curves corresponding to modules where the threshold is exceeded are displayed. From the statistical results, it can be clearly observed that the membrane potential fluctuation amplitude of the MLG1 model is significantly affected by changes in illumination. Its peak membrane potential differences and fluctuation frequency both exhibit noticeable irregular variations, indicating that the model is relatively sensitive to changes in light intensity. In contrast, the membrane potential time series of the proposed model maintains a consistently low fluctuation amplitude within the same range of illumination variation, with its variance values concentrated and showing no significant abnormal fluctuations. This result fully verifies the advantage of the proposed model in terms of illumination robustness. Embedding Daubechies wavelet into ON/OFF channels effectively suppresses the interference of illumination changes on neuronal responses, providing stable feature input for collision detection and spatial localization in dim light environments.

**Table 2:** Luminance values and contrast of experimental scenarios and alert frames.

Number	Imax	Imin	$C_{\mathrm{RMS}}$	$C_{M}$	Alert Frame of Proposed Model	Alert Frame of MLG1 Model
Figure 2 (1)	34	0	15.35	0.13	201	238
Figure 4 (1)	82	0	14.28	0.32	201	236
Figure 4 (2)	44	0	7.65	0.17	210	245
Figure 4 (3)	34	0	6.00	0.13	213	-
Figure 5 (1)	123	0	31.07	0.48	102	86
Figure 5 (2)	34	0	7.82	0.13	103	120
Figure 5 (3)	23	0	5.40	0.09	105	-
Figure 6 (1)	129	0	35.83	0.51	143	138
Figure 6 (2)	50	0	13.33	0.20	170	174
Figure 6 (3)	19	0	4.63	0.07	160	205
Figure 7 (1)	149	0	40.65	0.58	160	156
Figure 7 (2)	91	0	23.33	0.36	174	161
Figure 7 (3)	15	0	3.48	0.06	189	-

### 4.6. Comparison with Engineering Techniques

**Figure 9:**
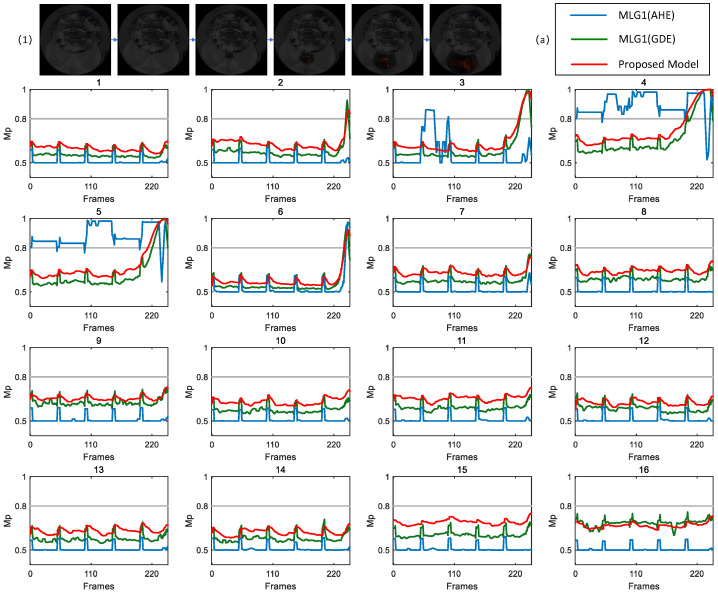
The proposed model has membrane potential responses exceeding threshold of 0.8 in five modules, indicating that the five modules are effectively involved in target motion recognition. In contrast, MLG1(AHE) exhibits membrane potential responses exceeding the threshold only in modules 3, 4, 5 and 6, suggesting that some functional modules in MLG1(AHE) do not function effectively. Additionally, MLG1(GDE) demonstrates membrane potential response characteristics identical to those of the proposed model.

**Figure 10:**
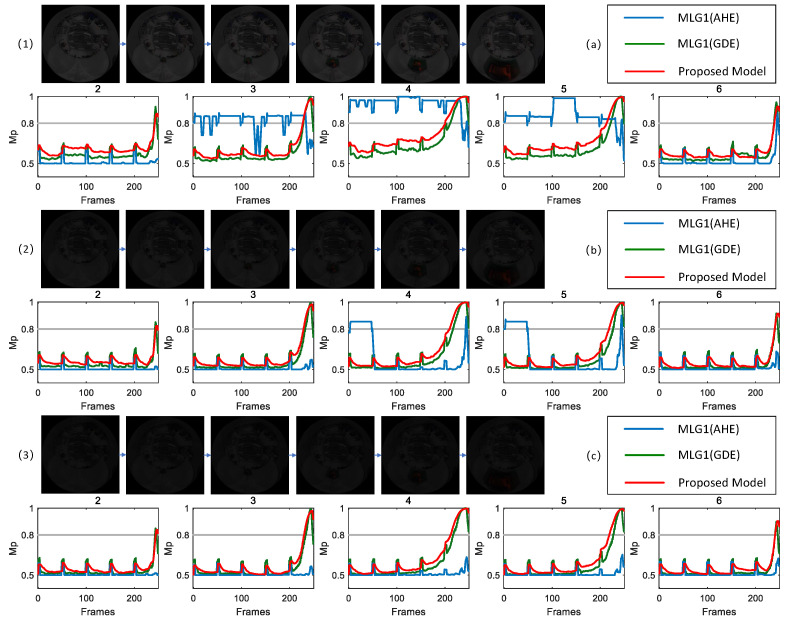
In three different lighting scenarios (with contrast $C_{M}$ of 0.32, 0.17, and 0.13), for MLG1 (AHE) at $C_{M}=0.32$, its membrane potential responses exceed threshold only in modules 3, 4, 5 and 6. When the contrast decreases to 0.13, none of the modules in MLG1(AHE) reach threshold. In contrast, under three different contrast conditions, both MLG1(GDE) and proposed model exhibit consistent membrane potential response characteristics.

**Figure 11:**
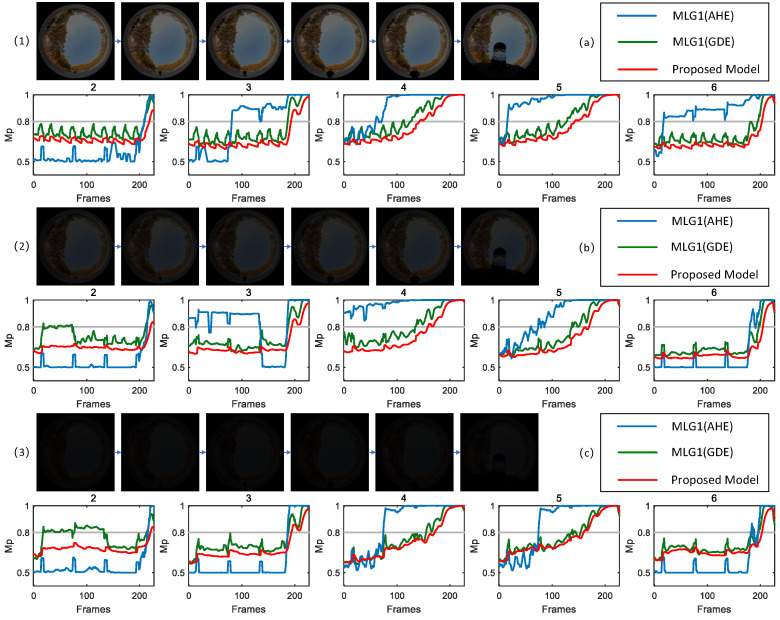
Tests are conducted in three outdoor dim light scenes with different illumination levels ($C_{M}=0.51$, 0.20 and 0.07, respectively). The MLG1(AHE) model generates membrane potential responses in five modules, but its target localization stability is relatively poor. For the MLG1(GDE) model, the core module that first produces a membrane potential response is inconsistent across three outdoor scenes with varying contrast levels. In contrast, the proposed model exhibits stable module activation in all three outdoor dim light scenes.

To validate robustness of the proposed model in dim light scenarios, we conduct comparative experiments with methods commonly used in traditional engineering practices. Specifically, adaptive histogram equalization (AHE) and gradient domain enhancement (GDE) are integrated into the proposed MLG1 model based on an ON/OFF mechanism. For ease of description and analysis in subsequent experiments, the model incorporating adaptive histogram equalization is denoted as MLG1(AHE), while the model integrating the gradient domain enhancement method is denoted as MLG1(GDE). Scenes with varying illumination levels are selected for testing and experimental results are shown in Figure 9, Figure 10 and Figure 11, respectively.

It is evident from the experimental results shown in Figure 9 that, in this dim light scenario, the proposed model exhibits membrane potential responses exceeding threshold of 0.8 in five core functional modules (i.e., modules 2, 3, 4, 5 and 6), indicating that all five modules effectively participate in target motion recognition. In contrast, MLG1(AHE) shows membrane potential responses exceeding threshold only in modules 3, 4, 5 and 6, while module 2 fails to reach threshold, suggesting that some functional modules in MLG1(AHE) do not function effectively. Notably, MLG1(GDE) demonstrates membrane potential response characteristics identical to those of the proposed model, with both consistently identifying module 4 as the core motion module of the object.

To further validate robustness of each model under dim light scenes with varying contrast levels, three distinct contrast scenarios (with contrast ratios $C_{M}$ of 0.32, 0.17 and 0.13) are set up for comparative experiments based on the scene corresponding to Figure 9. The experimental results are shown in Figure 10. From the experimental data and membrane potential curve characteristics presented in Figure 10, it is clearly observable that the performance of MLG1(AHE) model is significantly affected by contrast. At a higher contrast level ($C_{M}$ = 0.32), its membrane potential responses exceed the threshold only in modules 3, 4, 5 and 6, while module 2 remains inactive. When the contrast decreases to 0.13, none of the modules in MLG1(AHE) reach threshold, indicating a complete loss of ability to recognize moving objects. This demonstrates its poor adaptability in dim light scenes. In contrast, under three different contrast conditions, both MLG1(GDE) model and the proposed model exhibit consistent membrane potential response characteristics and both reliably identify module 4 as the core motion module of object, with no significant influence from variations in contrast.

After that, three outdoor dim light scenes with different illumination intensities ($C_{M}$ = 0.51, 0.20, 0.07, respectively) are selected for testing and experimental results are shown in Figure 11. From the results presented in Figure 11, it can be observed that in outdoor dim light scenarios, although MLG1(AHE) model generates membrane potential responses across all five core functional modules, thereby achieving module activation, the response intensity is unbalanced and the stability of target localization is relatively poor. The MLG1(GDE) model exhibits clear scene-dependent membrane potential response characteristics; across three outdoor scenes with varying contrast levels, the core module that first generates a membrane potential response is inconsistent, indicating insufficient response stability in complex outdoor dim light environments and difficulty in achieving continuous and stable target motion recognition. In contrast, the proposed model demonstrates stable module activation across three outdoor dim light scenes with different contrast levels, with response order of the core modules and target localization results remaining consistent, thereby exhibiting superior environmental adaptability and recognition stability.

## 5. Conclusions

This study addresses the critical challenge of bio-inspired MLG1 models in dim light environments, where photon shot noise typically corrupts spatiotemporal motion cues. Our primary objective was to develop an anti-noise computational framework by directly integrating multi-scale frequency domain decomposition and feature reconstruction of Daubechies wavelet into ON/OFF visual streams. The framework overcomes inherent limitations of dim light scenarios via a dual technical advantage: on the one hand, differential processing of ON/OFF mechanism demonstrates superior performance in low-signal-to-noise environments, avoiding the vulnerability of absolute luminance signals to noise interference. On the other hand, incorporation of Daubechies wavelet enables simultaneous noise suppression and enhancement of discriminative features, significantly improving the quality of feature inputs. Systematic experimental results demonstrate the numerical advantages of this approach, as the proposed model maintains reliable target localization at a contrast ratio as low as 0.06, significantly outperforming traditional engineering methods like AHE, which fail when the contrast drops to 0.13. Looking forward, low-power dedicated vision processing modules can be designed based on the computational logic presented here, with a focus on adapting to resource-constrained applications such as micro-mobile robots and portable environmental detection devices. This would address the existing challenge of balancing energy consumption and performance in dim light perception tasks on current hardware platforms.

## Figures and Tables

**Table 1:** Parameters of proposed model.

Equation	Parameters	Description
(7)	Δt=1	the duration of luminance change
(15) and (20)	σ∈[0,1]	standard deviation of Gaussian function in ON/OFF channels
(18) and (21)	ς=0.01	standard deviation of Gaussian function in ON/OFF channels
(22)	$\alpha_{1,2}=1,\alpha_{3}=0$	term coefficient in summation layer
(24)	μ=0.05	coefficient in sigmoid function
(25)	ν=4	scale coefficient in spiking
(25)	$T_{sp}=0.8$	spiking threshold
(26)	$t_{n}=\left[4,6\right]$	time window by discrete digital frames
(26)	$n_{sp}=\left[4,6\right]$	number of spikes within $t_{n}$

## Data Availability

The original contributions presented in this study are included in the article. Further inquiries can be directed to the corresponding authors.
